# Including Caregivers in the Community Aging in Place, Advancing Better Living for Elders Program

**DOI:** 10.1093/geroni/igaa057.3231

**Published:** 2020-12-16

**Authors:** Beth Fields, Caylee Yanes, Molly Ennis, Pamela Toto

**Affiliations:** 1 University of Wisconsin–Madison, Madison, Wisconsin, United States; 2 University of Pittsburgh, Pittsburgh, Pennsylvania, United States

## Abstract

Older adults frequently turn to informal caregivers for support to age independently in their home as long as possible. Yet, many evidence-based programs designed to support aging in place do not include caregivers, including the well-known Community Aging in Place, Advancing Better Living for Elders (CAPABLE) program. The purpose of this qualitative study was to adapt CAPABLE to include caregivers using a grounded theory approach. Data collection occurred with stakeholders from an Area Agency on Aging (AAA) in Pennsylvania. Two, 60-minute focus groups were conducted with frontline providers (n=7) and administrators (n=7). Eight, 30-minute individual interviews were conducted with AAA consumers including older adults and their caregivers. Constant comparative analysis of the data were completed using NVivo 12 Pro. Stakeholders described three considerations for adapting CAPABLE to include caregivers: older adult preference and caregiver willingness, clear guidelines and expectations, and hands-on training. Older adults and caregivers recognized the need to “allow them to decide when and why family should be involved in the program.” Frontline providers and administrators explained the importance of “determining whether older adults and caregivers should have shared or separate goals for the program.” All stakeholders expressed that including caregivers would “reaffirm the hands-on training like fall prevention.” These perspectives shed light on how and why to include caregivers in CAPABLE. Information gleaned from this study may help researchers think about ways in which to adapt other evidence-based programs to include caregivers, and help healthcare providers target and support caregivers in the delivery of their services.

